# The Prevalence of the Nasal Vestibular Body in Patients With Nasal Obstruction

**DOI:** 10.7759/cureus.56194

**Published:** 2024-03-14

**Authors:** Jose Luis Trevino Gonzalez, Aldo Sergio Fuentes Torres, Josefina Alejandra Morales Del Angel

**Affiliations:** 1 Otolaryngology - Head and Neck Surgery, Hospital Universitario Dr. José Eleuterio González, Monterrey, MEX

**Keywords:** concha bullosa, turbinate hypertrophy, septal deviation, nasal vestibular body, nasal obstruction

## Abstract

Background and aim

Nasal obstruction is one of the most common reasons for consultation addressed by otolaryngologists. There are anatomical, physiological, and pathological etiologies. Sometimes the treatment can become a challenge for the specialist, so a detailed evaluation of the etiologies must be carried out.

The involvement of the nasal vestibular body (NVB) in obstructive symptoms has been described. Therefore, we must be familiar with its anatomy, presentation, and contribution to this symptomatology. This study aimed to highlight the importance of NVB in the role of nasal obstruction and know the impact that it adds to the symptoms of patients through a validated instrument on their quality of life.

Material and methods

A retrospective, descriptive, and analytical study was conducted on 113 patients with nasal obstruction who attended the outpatient clinic of the Otolaryngology and Head and Neck Surgery Service in a tertiary-level hospital in Monterrey, Mexico from January 2021 to January 2023. The Nasal Obstruction Symptom Evaluation (NOSE) scale was applied to assess the impact of this symptom on the quality of life of the subjects.

The causes involved in the obstructive symptoms were identified by physical examination, including NVB. Two groups were made as follows: with the presence of the NVB and with the absence of the NVB, and the means of the NOSE scale were compared.

Results

A total of 113 patients were included, 59 male patients (52.20%) and 54 female patients (47.80%). The presence of NBV was found in 72 patients (63.70%). Other causes of nasal obstruction were found in 35 patients (31%), with chronic rhinitis being the most frequent in 27 subjects (23.90%). The mean NOSE scale score was higher in the group with the presence of the NVB than in the group with the absence of the NVB (p<0.05). The primary outcome of this study was to know the prevalence of NVB in the Hispanic population with nasal obstruction who attends the otolaryngology clinic. The secondary outcome was to know the relationship between the presence of NVB and the NOSE scores.

Conclusion

In this study, we observed that NVB is frequently present in patients with nasal obstruction in northeastern Mexico. There is an association between the presence of NVB and higher scores on the NOSE scale. New research will be needed to assess the effectiveness of NVB surgery in improving nasal obstruction and to determine the impact of NVB on nasal obstruction in isolation.

## Introduction

Nasal obstruction is one of the most common reasons for consultation addressed by otolaryngologists and it is associated with a decrease in quality of life [1]. Up to a third of the general population complains of this symptom and there is no gender predilection [2]. By definition, nasal obstruction is a perception of insufficient airflow or increased air resistance through the nose [3]. Etiologies can be anatomical, physiological, or pathological, but are more frequently multifactorial when more than one cause coexists. The consequences of nasal obstruction range from simple breathing discomfort to cognitive manifestations.

The nose plays a role in immune defense by acting as a filter to remove particle pollutants and humidification of inspired air, in addition to participating in olfaction [4]. The upper respiratory tract is lined by pseudostratified ciliated columnar epithelium that allows the aforementioned functions [5]. In the medical treatment of nasal obstruction, the aim is to reduce edema and inflammation of the mucosa with the use of intranasal steroids [6]. The surgical treatment can correct anatomical and pathological causes, for which a complete evaluation must be carried out based on intentional questioning and physical examination of causes of nasal obstruction. The importance of an accurate diagnosis results in optimal treatment, therefore all contributing causes to nasal obstruction must be known, including the nasal vestibular body (NVB), a recently described structure that is associated with these symptoms and for which a surgical reduction with radiofrequency has been described in selected cases [7].

In 2016, Locketz et al. first described NVB as a mound of soft tissue in the nasal vestibule, which may contribute to nasal obstruction in patients [8]. Previously, there was no knowledge regarding the existence of this structure, so its name was proposed as the nasal septal body [9]. In 2020, Ibrahim et al. described the participation of the NVB in nasal obstruction by comparing 18 patients with surgical reduction of the NVB against 10 patients with other nasal surgical interventions without reduction of the NVB, with improvement in the “SNOT 22” and “nasal breathing” scores in the first group [1]. In 2022, it was published by Vargas-Cárdenas et al. that computed axial tomography measurement of the NVB could correlate with symptoms of nasal obstruction [10].

It is important for the otolaryngologist to know the existence of NVB, integrate it into the nasal anatomy, and recognize its importance in obstructive symptomatology. This is the first study to determine the prevalence of NVB in the population of northeastern Mexico, given that these data are unknown and nasal obstruction is a frequent symptom.

## Materials and methods

Study subjects

A retrospective, descriptive, and analytical study was conducted on 113 patients with nasal obstruction who attended the outpatient clinic of the Otolaryngology - Head and Neck Surgery Department at the Hospital Universitario Dr. José Eleuterio González in Monterrey, Mexico from January 2021 to January 2023. The research protocol #OT24-00001 was approved by the Research and Institutional Ethics Committee of Hospital Universitario Dr. José Eleuterio González. The authors assert that all procedures contributing to this work comply with the ethical standards of the relevant institutional guidelines on human experimentation and the Helsinki Declaration of 1975, revised in 2008. As a confidentiality mechanism the full names of the research subjects were not used, only the registration or folio number assigned at the time of inclusion in the protocol was used.

NOSE scale application

The Nasal Obstruction Symptom Evaluation (NOSE) scale is a tool validated in 2004 to subjectively measure nasal obstruction, which was applied to understand the impact of nasal obstruction on their quality of life [11]. The scale has five items (nasal congestion or poor ventilation, nasal blockage or obstruction, problem breathing through the nose, problem sleeping, inability to get enough air through the nose during exercise or effort), each item scored by the patient with a score from 0 to 4 (0=not a problem, 1=very mild problem, 2=moderate problem, 3=fairly bad problem, 4=severe problem), resulting in a total sum ranging from 0 to 20. Additionally, this sum can be expressed as a percentage. This scale was applied to all patients with complaints of nasal obstruction.

Physical examination

The physical examination was performed through anterior rhinoscopy to identify the cause of nasal obstruction. Chronic rhinitis was defined as inflammation of the nasal mucosa characterized by the following two or more symptoms: nasal congestion, anterior or posterior rhinorrhea, and sneezing or itching, at least one hour a day and for more than 12 weeks a year. Two groups were obtained as follows: with the presence of NVB and with the absence of NVB.

Simple computed axial tomography of the paranasal sinuses was evaluated in the patients who were requested. The images were obtained through an institutional platform of the Hospital Universitario Dr. José Eleuterio Gonzalez from the Radiology Department. Images were gathered using a 64-slice CT (GE LightSpeed VCT; Waukesha, WI: GE Medical Systems) with a voltage of 120 kV, effective mAs of 18, and a field of view of 142x278 mm. Obtained images contained 1.25 mm axial slices and coronal and sagittal reconstructions in a high-definition bone window. The images were evaluated by the authors of this study.

Patients

A sample of 113 subjects were estimated in the study, using a formula for estimating a proportion in an infinite population [N=(Za)^2^ (p)(q)/δ^2^] with the primary objective of determining the prevalence of NVB in a population with nasal obstruction, expecting a proportion of 3% presence of NVB in patients with nasal obstruction, with a bilateral significance of 2% and a power of 99%.

Inclusion and exclusion criteria

The patients were selected by focusing on functional adult individuals between 18 and 65 years of age, male or female, with complaints of nasal obstruction and capable of describing this symptom in order to rule out other nasosinusal pathologies. The exclusion criteria were cognitive impairment, to avoid bias when applying the NOSE scale; previous nasal surgery, which could have been surgical treatment for nasal obstruction before; previous nasal endoscopic surgery, coexistence of benign or malignant tumors in paranasal sinus or nasal cavity, nasal fracture, coexistence of infectious pathology (common cold, influenza, COVID-19 or rhinosinusitis); and pregnancy, because during this period hormones can increase the symptoms of nasal obstruction.

Statistic analysis

The data described in the variables section were emptied into an Excel sheet (Redmond, WA: Microsoft Corp.) without identifying data. The Excel sheet was transferred to SPSS (Armonk, NY: IBM Corp.). The statistics were analyzed with the SPSS program. The numerical variables were described with measures of central tendency and dispersion and the categorical variables were described using absolute numbers and frequency percentages.

The association between categorical variables was analyzed using the chi-square test. The association between categorical and numerical variables of parametric distribution was evaluated with the Student's t-test and ANOVA, in case of two or more than two groups, respectively.

In case of non-parametric distributions, the relationship between numerical and categorical variables was evaluated with the UMW or Kruskal Wallis test, in case of two or more than two groups, respectively. Finally, the correlation between two numerical variables was evaluated with the Pearson correlation coefficient. P<0.05 was considered statistically significant.

## Results

Demographics

A total of 113 patients were included, 59 male patients (52.20%) and 54 female patients (47.80%). The mean age and its standard deviation (SD) were 41.35 years and 15.07 years, respectively. The demographic data and causes of nasal obstruction are detailed in Table 1. The most common age range was 18-30 years, with 38 subjects (33.60%).

**Table 1:** Demographic data and causes of nasal obstruction. SD: standard deviation; NVB: nasal vestibular body

Variable	Total
Male, n	59
Female, n	54
Age, mean (SD)	41.35 (15.07)
Age range	
18-30, n (SD)	38 (33.60)
31-40, n (SD)	13 (11.50)
41-50, n (SD)	24 (21.20)
51-60, n (SD)	27 (23.90)
61-65, n (SD)	11 (9.70)
NVB	
Presence, n (SD)	72 (63.70)
Absence, n (SD)	41 (36.30)
Other causes of nasal obstruction	
Yes, n (SD)	35 (31.00)
No, n (SD)	78 (69.00)
Distribution of other causes of nasal obstruction	
Concha bullosa, n (%)	1 (0.90)
Septal deviation, n (%)	6 (5.30)
Turbinate hypertrophy, n (%)	1 (0.90)
Chronic rhinitis, n (%)	27 (23.90)

Findings through anterior rhinoscopy and computed axial tomography of the paranasal sinuses

**Figure 1:**
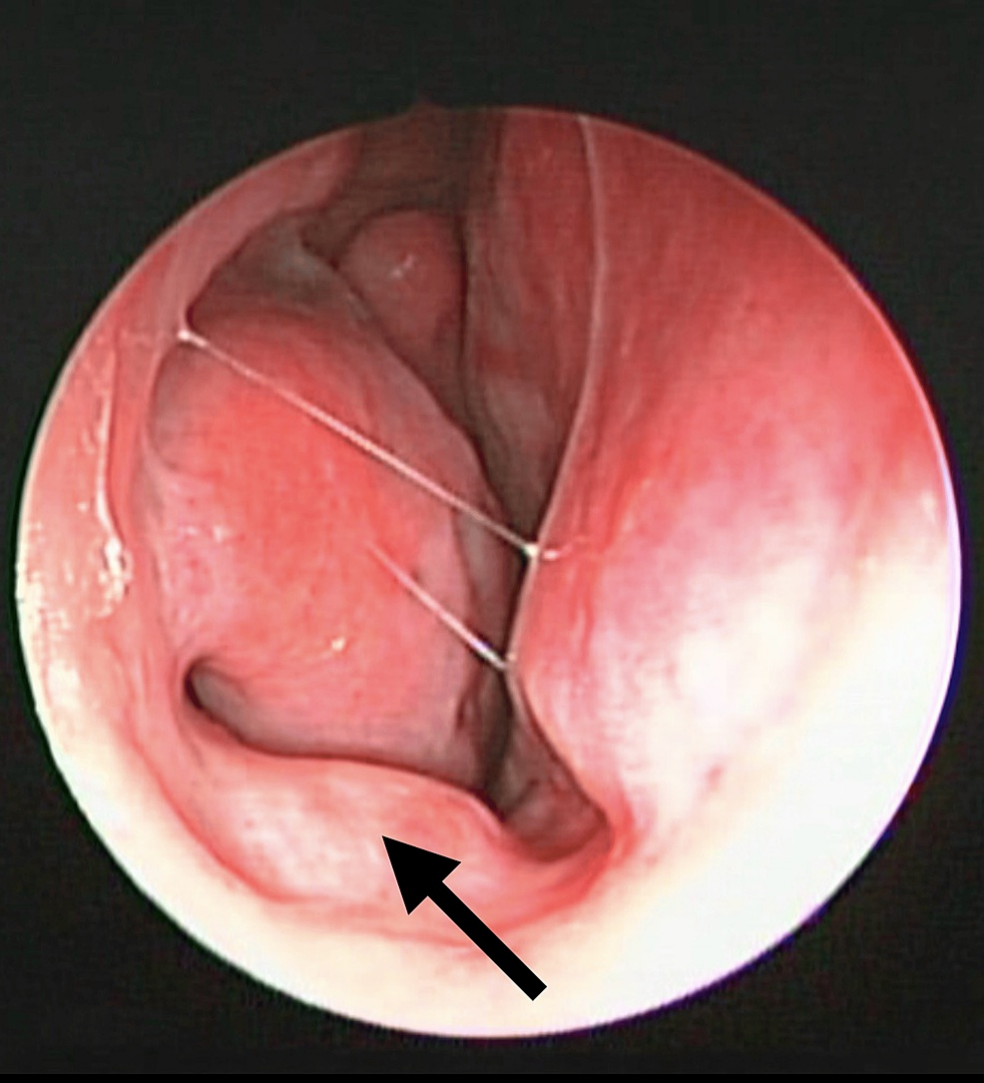
Nasal endoscopy with a zero-degree view in a patient with a complaint of chronic nasal obstruction in right nostril. The arrow shows the right nasal vestibular body (NVB).

**Figure 2:**
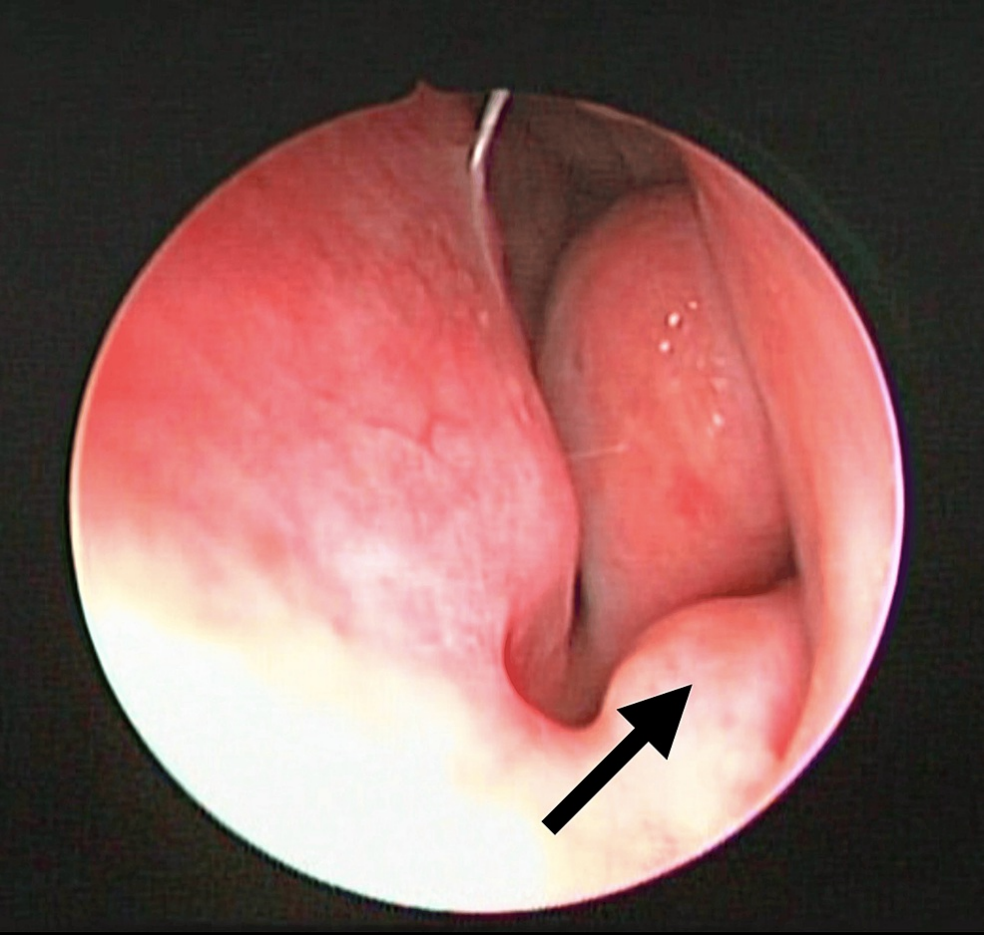
In the same patient, the NVB can be observed in contralateral side at the level of the internal nasal valve. The arrow shows the left NVB. NVB: nasal vestibular body

**Figure 3:**
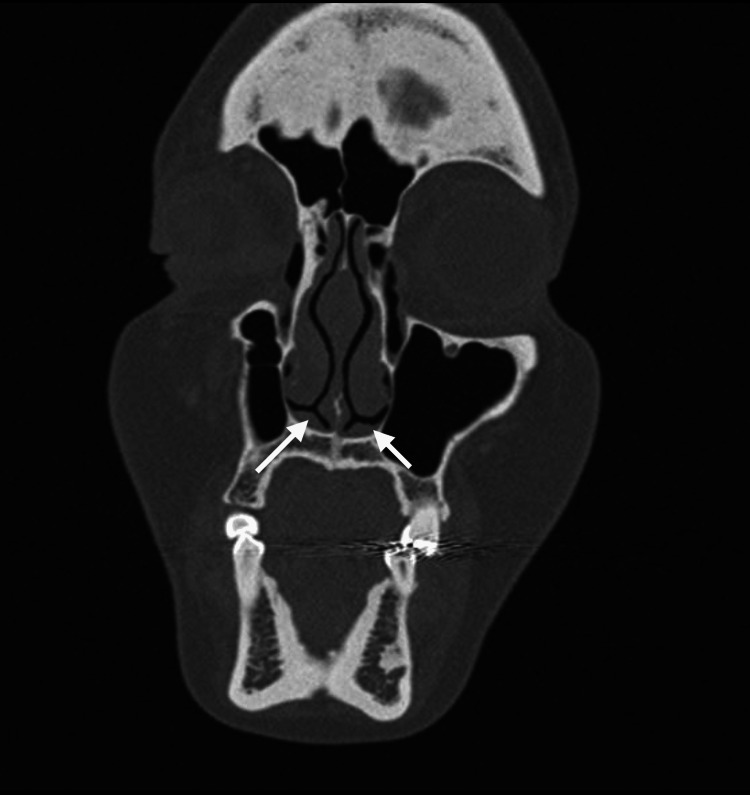
Coronal view of paranasal sinus computerized tomography. The NVB can be seen in both nostrils at the level of internal nasal valve. Arrows show NVB. NVB: nasal vestibular body

The presence of NVB was found in 72 patients (63.70%). Other causes of nasal obstruction were found in 35 patients (31%), with chronic rhinitis being the most frequent in 27 subjects (23.90%) and other less common causes were concha bullosa, septal deviation, and turbinate hypertrophy. Bilateral NVB distribution was the most common in 32 patients (Figures 1-3). The coexistence of NVB with other causes of nasal obstruction occurred in 27 of 72 patients (23.9%) (Table 2).

**Table 2:** Presentation of NVB and other causes of nasal obstruction. NVB: nasal vestibular body

Variable	Male, n=59 (52.2%)	Female, n=52 (47.8%)	Total, n=113 (100%)
NVB, n (%)	36	36	72 (63.70%)
Right, n (%)	8	12	20 (17.69%)
Left, n (%)	11	9	20 (17.69%)
Bilateral, n (%)	17	15	32 (28.31%)
Other causes of nasal obstruction, n (%)	21	14	35 (31%)
Septal deviation, n (%)	3	3	6 (5.30%)
Turbinate hypertrophy, n (%)	0	1	1 (0.88%)
Chronic rhinitis, n (%)	15	12	27 (23.90%)
Concha bullosa, n (%)	0	1	1 (0.88%)
Coexistence of NVB and other causes of nasal obstruction, n (%)	17	10	27 (23.90%)

NOSE scale

The results of the NOSE scale are reported in Table 3 and Table 4 by individual item (0-4 points), by sum of the five items (0-20 points), and as a percentage (0-100 points). When analyzing patients with the presence and absence of NVB, no statistically significant difference was found in the proportion of sex or age between both groups. The mean NOSE score of each item, total items, and total percentage of each group with the presence and absence of NVB are shown in Table 4, which were higher in the group with the presence of the NVB than in the group with the absence of the NVB. All these differences were statistically significant (p<0.05).

**Table 3:** Results of the NOSE scale of the 113 patients. NOSE: Nasal Obstruction Symptom Evaluation

Variable	Average (SD)
Nasal congestion or poor ventilation (0-4)	2.17 (1.09)
Nasal blockage or nasal obstruction (0-4)	1.88 (1.18)
Problem breathing through the nose (0-4)	1.92 (1.13)
Sleeping problem (0-4)	2.17 (1.20)
Inability to get enough air through the nose during exercise or exertion (0-4)	1.99 (1.29)
Total items (0-20)	10.16 (4.43)

**Table 4:** Association between the NOSE scale score per individual item, total items (0-20), total percentages, and presence or absence of the NVB. *Statistical significance was set at p<0.05. NVB: nasal vestibular body; NOSE: Nasal Obstruction Symptom Evaluation

Variable	NVB present (average)	NVB absent (average)	p-Value
Nasal congestion or poor ventilation (0-4)	2.43	1.73	0.001*
Nasal blockage or nasal obstruction (0-4)	2.25	1.24	˂0.001*
Problem breathing through the nose (0-4)	2.23	1.39	˂0.001*
Sleeping problem (0-4)	2.45	1.68	0.001*
Inability to get enough air through the nose during exercise or exertion (0-4)	2.36	1.34	˂0.001*
Total items (0-20)	11.73	7.41	˂0.001*
Total percentages	58.68	37.07	˂0.001*

The correlation between the age of each item on the NOSE scale, the total of each item, and the percentages was analyzed. There was no statistically significant correlation between age and these values. When analyzing the difference in the score of each NOSE item, total items (0-20), and total percentages between the previously described age ranges in years (18-30, 31-40, 41-50, 51-60, 61-65), there was no statistically significant difference between the age ranges.

## Discussion

Nasal obstruction is a common symptom in the otolaryngologist's office, with an immense range of etiologies, which can coexist and contribute together to the symptomatology. Physical examination is essential to identify the cause of the obstruction, along with ancillary studies such as nasal endoscopy and sinus tomography. The anatomical etiologies are septal deviation, turbinate hypertrophy, collapse of the internal nasal valve, concha bullosa, and adenoid hypertrophy. The nasal cycle is a physiological cause that is regulated by the parasympathetic nervous system increasing vascular congestion of the turbinates and allowing the inspired air to be conditioned; this contributes to airflow resistance and causes symptomatic obstruction [2]. The pathological causes may include polyps, benign and malignant tumors, and chronic rhinitis, defined as symptomatic inflammation of the mucosa that lasts more than 12 weeks [12]. The nasal mucosa plays an immunological role thanks to its epithelium which acts as a physical barrier against microorganisms and inhaled particles [13].

Nasal obstruction can be assessed by objective and subjective methods. Rhinomanometry is an objective way of measuring pressure and flow during respiratory cycles [7]. Subjectively, the application of scales allows us to know the degree of repercussion of this symptomatology referred by the patient. The NOSE scale is a tool validated in 2004, which evaluates the impact of nasal obstruction on quality of life and the severity can be classified according to the total score obtained as mild obstruction (5-25 points), moderate obstruction (30-50 points), severe obstruction (55-75 points), and extreme obstruction (80-100 points) [14].

The treatment of nasal obstruction is directed according to the cause and consists of medical treatment, surgery, or both. Nasal steroids act at the level of the nasal mucosa, decreasing the chemotaxis of neutrophils and eosinophils, with a reduction in the release of mediators from mast cells and basophils and, ultimately, reducing edema and inflammation. Their safety and efficacy profile make it a first-line treatment for most cases [15]. The results of the treatment of nasal obstruction can be unsatisfactory, becoming a challenge for the specialist, which leads to considering other available options in the management of obstructive symptoms and make sure you identify the causes.

In this study, nasal obstruction occurs in a similar way in both males and females (59 and 54 subjects, respectively), being more common between 18 and 30 years of age, supporting the idea that there is no gender predilection (Table 1). NVB was found in 72 of the 113 patients evaluated. The proportion of males and females was not statistically significant, as similar proportions were obtained. In 27 of 72 patients, another cause of nasal obstruction was found, of which chronic rhinitis was the most frequent finding in 21 patients (29.16%), being able to associate the probable inflammatory state of the nasal mucosa with the presence of the NVB.

Locketz et al. described this structure first in 2016, as a soft tissue mass in the nasal vestibule, which goes unnoticed and can contribute to nasal obstruction [8]. This study allowed the incorporation of NVB into the nasal anatomy and analyzed its physiological contribution. In patients with persistent obstructive symptoms even with medical treatment, they may benefit from surgical reduction alone or in conjunction with other nose procedures. This structure responds to chemical reduction with nasal decongestants, which sheds light on its role in nasal physiology. It is covered with mucosa and its location within the internal nasal valve provides one of the first lines of defense against microorganisms and particles. This erectile tissue responds to parasympathetic control stimuli, promoting the secretion of mucus, so its function may be associated with air filtration and humidification. In this study, we found NVB in the floor at the level of the internal nasal valve, adding as a finding that the presentation can be unilateral or bilateral and with different volumes or degrees of hypertrophy, not always being symmetrical in cases of bilateral presentation.

With computed axial tomography, it is possible to demonstrate NVB (Figure 3). Its measurement by this imaging method may be possible, in addition to finding a relationship with the size and symptoms of nasal obstruction [10]. In this study, we did not measure the NVB by this imaging method.

Ibrahim et al. in 2020 performed a retrospective study in which they compared the surgical reduction of the NVB with other interventions to relieve the obstructive symptoms and other interventions without reduction of NVB, with improvement in the “Sino-nasal Outcome Test (SNOT-22)” and “nasal breathing” scores [1]. Compared to our study, the SNOT-22 scale was not applied, since it evaluates items related to rhinosinusitis. Instead, the NOSE scale was used to evaluate the impact on the quality of life of patients with nasal obstruction and to understand their perception when performing activities such as sleeping and physical effort. We found that the importance of evaluating the quality of life of patients with obstructive symptoms lies in understanding the impact on the limitation of daily activities and can be implemented within the medical interview through the application of scales. The advantage of using scales is that they can be easily reproduced in a serial manner to evaluate follow-up and assess the response to treatment. The mean NOSE scale score was higher in the group with NVB presence than in the group with NVB absence (p<0.05) (Table 4).

The prevalence of NVB is frequent in our environment and its anatomical location provides the opportunity to easily identify it in physical examination, nasal endoscopy, or even in computed tomography of the paranasal sinuses, where the dimensions of this structure can be measured, and this could correlate with symptoms of nasal obstruction [9]. The recognition of NVB and its association with symptoms of nasal obstruction in the studies carried out to date has allowed us to consider the option of a surgical approach to this structure in well-selected patients, whose symptoms are mainly caused by its presence. With this background, it will be possible to carry out intervention studies with larger populations than existing studies. Some techniques include tissue reduction [6], which can be achieved by radiofrequency ablation [10].

The generation of statistics is important, especially prevalence, since it allows classification and analysis to be carried out with the information obtained. The relevance of this study lies in knowing the prevalence of this structure in patients with nasal obstruction, since it is one of the main reasons for consultation with the otorhinolaryngologist. This is the first study that highlights these data, which will contribute to the generation of statistics on Hispanic patients.

It will also be important to know the prevalence in asymptomatic patients in order to have broader and more accurate statistics. This study has limitations since the evaluation of the symptom was subjective, applying a scale that assesses the impact on the patient's daily activities. The coexistence of other causes of nasal obstruction in patients with the presence of NVB, such as chronic rhinitis in 29.16%, can considerably increase the NOSE scale score. It will be necessary to carry out studies that reveal the participation of only NVB in nasal obstruction, without other causes of nasal obstruction. In addition, it will be necessary to know the prevalence in the general population and not only with nasal obstruction.

## Conclusions

The NVB is frequently found in patients with nasal obstruction in northeastern Mexico. It is important for the otolaryngologist to carefully evaluate the causes involved in nasal obstruction and know how to recognize them, including NVB. The application of instruments that evaluate the impact on quality of life, such as the NOSE scale, is essential to provide adequate follow-up and monitor the behavior of symptoms with a treatment granted. Further research will be needed to evaluate the effectiveness of NVB surgery in improving nasal obstruction and to determine its contribution to symptomatology in isolation.

## References

[1] Ibrahim N, Tyler MA, Borchard NA, Rathor A, Nayak JV (2020). Nasal vestibular body treatment for recalcitrant nasal obstruction. Int Forum Allergy Rhinol.

[2] Hsu DW, Suh JD (2018). Anatomy and physiology of nasal obstruction. Otolaryngol Clin North Am.

[3] Corredor-Rojas G, García-Chabur MA, Castellanos J, Moreno S, Pinzón M, Peñaranda A (2021). Nasal obstruction and quality of life assessment after septoplasty with turbinoplasty: correlation between subjective scales. Am J Rhinol Allergy.

[4] Patel RG (2017). Nasal anatomy and function. Facial Plast Surg.

[5] Bizaki AJ, Numminen J, Taulu R, Kholova I, Rautiainen M (2016). Treatment of rhinosinusitis and histopathology of nasal mucosa: a controlled, randomized, clinical study. Laryngoscope.

[6] Leader P, Geiger Z (2023). Vasomotor rhinitis. StatPearls [Internet].

[7] Yang A, Kim D, Tsai EF, Chang MT (2022). The nasal vestibular body and its role in nasal obstruction. Curr Otorhinolaryngol Rep.

[8] Locketz GD, Teo NW, Walgama E, Humphreys IM, Nayak JV (2016). The nasal vestibular body: anatomy, clinical features, and treatment considerations. Eur Arch Otorhinolaryngol.

[9] Meng X, Zhu G (2021). Nasal septal swell body: a distinctive structure in the nasal cavity. Ear Nose Throat J.

[10] Vargas-Cárdenas LG, Ramírez-Oropeza FJ, Gómez-Monterrosas O, Lugo-Machado JA (2022). Tomographic dimensions of the nasal vestibular body in relation to nasal obstruction in adults. Romanian J Rhinol.

[11] Standlee AG, Hohman MH (2017). Evaluating the effect of spreader grafting on nasal obstruction using the NOSE scale. Ann Otol Rhinol Laryngol.

[12] Meng Y, Lou H, Wang Y (2019). Endotypes of chronic rhinitis: a cluster analysis study. Allergy.

[13] Freeman SC, Karp DA, Kahwaji CI (2023). Physiology, nasal. StatPearls [Internet].

[14] Lipan MJ, Most SP (2013). Development of a severity classification system for subjective nasal obstruction. JAMA Facial Plast Surg.

[15] Cox DR, Wise SK (2018). Medical treatment of nasal airway obstruction. Otolaryngol Clin North Am.

